# The “Survival Pending Revolution” COVID-19 vaccination campaign: an example of critical communication theory in action

**DOI:** 10.3389/fpubh.2023.1134104

**Published:** 2023-06-19

**Authors:** Dean Schillinger, Gabriel Cortez, Michelle Lee

**Affiliations:** ^1^Health Communications Research Program, University of California, San Francisco, San Francisco, CA, United States; ^2^Youth Speaks, Inc., Oakland, CA, United States

**Keywords:** health equity, health communication, public health, communication theory, health literacy, COVID vaccination, structural determinants of health

## Abstract

**Introduction:**

We carried out a two-phase, qualitative evaluation of a novel public health campaign to promote COVID-19 vaccination among youth and young adults of color (YOC), called Survival Pending Revolution. The campaign, commissioned by California's Department of Public Health, was created by YOC spoken word artists, under the direction of the organization, Youth Speaks.

**Methods:**

In phase 1, we describe the communication attributes of the campaign's nine video-poems, coded the content of the pieces, and applied thematic analysis to describe the themes conveyed. In phase 2, we carried out a comparative health communication study to assess the content's potential value. We exposed a sample of the target audience (YOC) to the content of Survival Pending Revolution and a widely viewed comparator campaign (The Conversation). Using a focus group, we solicited participants' views using a semi-structured approach. Using thematic analysis, we summarized the reactions that arose when participants reflected on the attributes of each campaign.

**Results:**

Findings from phase 1 reveal how engaging YOC artists who embrace Youth Speaks' philosophy of harnessing “life as primary text” resulted in content that is aligned with critical communication theory, focusing on structural determinants of health, including themes of overcoming oppressive systems, health and social inequities, and medical discrimination and mistrust. Findings from phase 2 reveal that this arts-based campaign based on such critical communication theory, when compared to a more traditional campaign, promotes message salience, fosters emotional engagement, and provides a form of validation among historically oppressed groups such that they may be more open to, and potentially act on, the COVID-19 vaccination communications to which they are exposed.

**Discussion:**

As an example of critical communication, the Survival Pending Revolution campaign encourages health-promoting behavioral decisions while calling out the structural determinants of health that shape risks of exposure and constrain free choice. Engaging uniquely gifted members of marginalized populations as creators and messengers of campaigns lead to content that is aligned with a critical communication approach, whose goal is to aid disparity populations in both resisting and navigating systems that continue to locate them on the margins of society. Our evaluation of this campaign suggests that it represents a promising formative and interventional approach to engendering trust in public health messaging and promoting health equity.

## Introduction

In response to the relatively low COVID-19 vaccination rates observed among people of color (POC) in California—and especially among youth and young adults of color (YOC) (1)—California Department of Public Health (as part of its *Vaccinate All 58* initiative) granted awards to selected community-based organizations to develop and disseminate novel communication content to increase vaccination rates. In the spring of 2022, *Youth Speaks*, a youth arts and empowerment organization (www.youthspeaks.org), was granted funds to support the development and dissemination of a novel COVID-19 vaccination campaign, which it entitled *Survival Pending Revolution* (see campaign content at www.thebiggerpicture.org).

The resulting nine short videos that comprise the heart of the campaign have many attributes that distinguish them from more traditional public health communication campaigns. In brief, these include (1) the use of spoken word art as the medium for communication about COVID-19 vaccination; (2) the featuring of young (non-celebrity) individuals as public health spokespersons; and (3) the application of a novel approach to public health communication that is most closely aligned with what is known as critical communication theory (2).

The so-called critical approaches to health communication are concerned with how power influences society's cultural constructions of health and responses to illness (3). As Kline and Khan stated, “Critical communicators are motivated by an explicitly political and ethically grounded goal of fostering social justice, equity and human rights, achieved by unmasking the sociopolitical forces that regulate and constrain the health and illness experiences of various disadvantaged, marginalized, and/or oppressed groups of people”. As Zoller and Kline explained (2), “Critical theorizing involves deconstructing dominant, taken-for-granted assumptions about health, often with the hope of introducing possibilities for alternative, more inclusive meaning systems”. Table 1 compares and contrasts the attributes of traditional public health communication content with content often contained in communications that align with critical communication theory.

**Table 1:** Common features of public health communications when using traditional vs critical approaches.

**Attribute**	**Approach**	
	**Traditional**	**Critical**
Messenger	Experts; celebrities	“Regular”, everyday but compelling people
Strategy	Fact-based; fear-based	Contextualize problems; elucidate complexity; grounded in reality (“lived experience”)
Form	Logic-argument (cognitive)	Narrative (emotional)
Perspective/Positionality	Authoritative; unambiguous; directive	Questioning/critical; ambiguous/ambivalent; non-directive
Setting	Unique, exceptional, dramatized	Everyday
Genre	Educational, instructional	Creative: art, poetry, film

While critical communication theory has been discussed in academic circles (2–4), very little work has been done to highlight its execution in the real world. In addition, to the best of our knowledge, no research has evaluated the real-world effects on target audiences of critical communications relative to more traditional, state-of-the-art public health communication approaches. We conducted a preliminary, two-phase qualitative evaluation of a novel campaign related to COVID-19 vaccination from the perspective of critical communication theory and discuss the implications of our results for future campaigns by public health entities.

## Materials and methods

### Genesis of the campaign

Over the last 11 years, *Youth Speaks* (www.youthspeaks.org) has collaborated with the University of California San Francisco (UCSF) Health Communications Research Program at San Francisco General Hospital to support the creation of novel public health communication content, promoted under the label of *The Bigger Picture*. *Youth Speaks* supports the activation and application of YOC voices to issues of social change, leveraging high-quality live and digital artistic presentations using the spoken-word art form to help catalyze culture change and shift the behavior of people, institutions, and systems to benefit YOC. Although the creative process in *The Bigger Picture* is curated by experienced spoken-word poet mentors and public health communication experts, the creative work itself is generated by YOC poets. The resulting campaigns harness authentic YOC voices (i.e., voices that have not been filtered or recast by marketing departments), delivered *via* spoken-word poetry and film. Merging artistic expression with public health messaging, *The Bigger Picture* campaigns aim to shift the conversation from solely encouraging individual behavior change to inspiring youth and youth stakeholders to act for health justice by reversing the social, environmental, and structural forces that determine and perpetuate disease risk. As such, communications released under *The Bigger Picture* label represent public health literacy campaigns (5).

The process that *Youth Speaks* and its UCSF partner use to support the creation of public health literacy campaigns by YOC is well-established and has been previously described, along with the conceptual framework and logic model underlying this work (6–8). *The Bigger Picture* pedagogy is aligned with Brazilian Paolo Freire's seminal work, *Pedagogy of the Oppressed*, (9) and that of his compatriot Augusto Boal's related work in performance art, *The Theater of the Oppressed* (10). Boal describes the *Theater of the Oppressed* as “always seeking the transformation of society in the direction of liberation of the oppressed. It is both action in itself, and a preparation for future actions … it is not enough to interpret reality; it is necessary to transform it!” (10).

In the spring of 2022, two *Youth Speaks* poet-mentors conducted 2-week-long workshops with YOC artists from across the state who were commissioned to create spoken-word pieces whose intent was to foster productive conversation and motivation related to COVID-19 vaccination in YOC. As with prior *The Bigger Picture* workshops, these workshops offer (1) public health content (2), open discussions (3), writing exercises, and (4) feedback on drafts. Based on Freire and Boal's participatory pedagogy, *Youth Speaks* workshops encourage youth to view their “life as primary text”, i.e., to tap into their lived experiences when creating poetry. As the workshop process ensued, many participants articulated ambivalence around the idea of focusing on vaccination as the solution to the COVID-19 crisis. Ultimately, they landed on a common theme that seemed to animate their poetry: “Survival Pending Revolution”. This phrase, lifted from a quote by Huey P. Newton, co-founder of the Black Panther Party, became the title for the resulting COVID-19 vaccine campaign. During the civil rights movement in the US in the 1960s, the Black Panthers set up programs to provide basic assistance (food and medical care) to needy members of the Black community. Newton, who advocated for the need for transformative social change at any cost, stressed that “These programs satisfy the deep needs of the community, but they are not solutions to our problem. That is why we call them survival programs, meaning survival pending revolution” (11).

### Campaign content

In all, 15 original pieces were created by 14 poets. At the time of our evaluation, nine of these pieces had been turned into short films (4–6 min each) which were disseminated *via* social media in June, July, and August of 2022.

In the accompanying Tables 2–10, we provide links to, and summaries of, each video-poem, and include representative excerpts (N.B. All *Survival Pending Revolution* videos are bookended by the following introductory and concluding statements that establish their alignment with critical communication principles: “COVID is a virus that doesn't discriminate, but society does. People of color have suffered and died from COVID more than others. Now we have a vaccine...”).

**Table 2:** *A walk through the valley*, by Nia Lundkvist.

https://www.youtube.com/watch?v=fzOmA40Vge0&t=1s	
This piece tells the story of Nia's conflicting thought processes and ambivalent emotional journey as leading up to, and including, her COVID-19 vaccination. In the telling of this story, she both articulates the compelling need for protection against COVID-19 in her community due to structural racism, while also enumerating the ways in which medical racism and mistrust undermine her faith in the vaccine, making her decision-making process complex and her experience receiving the vaccine harrowing. In the poem she models bravery on behalf of her family and community in the face of uncertainty.	
Excerpt: *I can't help but replay When health officials first announced: “New vaccine!” The name Henrietta Lacks still echoes twice as loudly back. Thunderclap, Tuskegee experiment, ten times tightening a chest, Already hypertension-prone. Centuries of forced sterilization, And false beliefs of a higher pain tolerance, Still ongoing and still shrieking silently Underneath my skin as it prepares to be pierced. I know American healthcare To be one of many aliases for systems that don't live up to their name. My health: not its objective. My care: not the central priority at stake… What exactly is in that syringe? What are its long-term effects? How can I trust it, When I don't trust the system it came from?*	
**Attribute**	**Execution**
Messenger	Everyday, young Black woman
Strategy	Contextualize availability and receipt of vaccine in history of medical racism; meditation on moving through when faced with ambivalence and uncertainty
Form	First-person narrative
Perspective/positionality	Questioning, critical, non-directive, ambivalent
Setting	Hospital/vaccine site
Genre	Spoken word film
Main message	We must make difficult, brave choices (in this case to get the COVID vaccine) in the face of genuine uncertainty so as to be alive to fight the larger communal fight against racism and inequity

**Table 3:** *Cuantificando Lo Esencial* (*Quantifying the Essential*), by Sandra Vasquez.

https://www.youtube.com/watch?v=lHMTWXCJ6KA&t=1s	
Sandra tells the story of her mother and aunt, both essential (likely undocumented) workers who are continually exposed to COVID-19 as a result of their jobs. In the piece, she acknowledges the unique vulnerability of her family and community with respect to COVID-19 and a host of other unhealthy exposures, while also celebrating the resilience of her family and community to survive. Her naturalistic poem includes an open acceptance of the COVID-19 vaccine while also calling out the need for larger, systemic solutions that go beyond a vaccine. Excerpt: Original Spanish version: *Mi titi es una de pocas personas bilingües asignadas al sitio de vacunación Masivo en medio de una comunidad migrante Donde más de mil personas se vacunan dia por dia Titi sale a trabajar Con manos abiertas, listas para atender Las preguntas y ansiedades Que tengan la gente que ella sirve La gente obrera Indocumentada Inmigrante Sabemos que la vacuna Es un recurso, más no una solución Para la frontera que envuelve nuestros pulmones La vacuna es un exhalo temporal La vacuna es un resguardo para el momento La vacuna nos da espacio para respirar con Poquita más confianza La vacuna es una arma Cuyo defensa nos puede servir Para combatir este virus Y nombrar nuestras familias y comunidades necesarias*	
English translation: *My auntie is one of a few bilingual people assigned To a massive vaccine site for migrant communities Where more than a thousand people get vaccinated daily. My auntie leaves for work With open hands, ready to address All the questions and anxieties That the people she serves have. These are working people. Undocumented, immigrants. We know that the vaccine is a resource, but not a solution Against the border that encircles our lungs. The vaccine is a temporary exhale, The vaccine is a momentary defense. The vaccine gives us space to breathe With just a little more confidence. The vaccine is a weapon Whose defense serve us In fighting this virus, Naming our families and communities as “essential.”*	
**Attribute**	**Execution**
Messenger	Everyday young Latinx woman from low-income family
Strategy	Positions the vulnerability of undocumented Latinx families due to structural discrimination in direct contrast to their resilience and ability to survive. Positions a family member as someone who administers the vaccine to her community.
Form	Third-person narrative, family saga
Perspective/positionality	Critical, contextual, implied directive
Setting	Naturalistic: essential workplace, home
Genre	Spoken word film
Main message	We receive the COVID vaccine because we must (we are vulnerable to infection due to discriminatory policies), and we take agency in the process. This fortifies our resilience to enable us to combat structural violence

**Table 4:** *When nanny comes home from the hospital*, by Zoe Dorado.

https://www.youtube.com/watch?v=gQz8aEmbUMk&t=11s	
In this piece, a Filipina youth describes her feelings and concerns about her mother, a nurse who is continually exposed to COVID-19 at work. The piece honors the service of essential workers, especially the Filipino immigrant healthcare workforce, while calling out the unfair health consequences that can result from such exposures. The poem focuses on the emotional relief Zoe feels when she learns that her mother is now protected because of the new vaccine. Excerpt: *What greater achievement than leaving your archipelago Pipelined halfway across the ocean, Pushed and pulled by two countries, Got caught in the middle of their economy. But, how lucky to be part of a healthcare system That sees you as expendable, I mean essential, I mean That Filipinos make up 4% of the nurses in the US, But 31.5% of the nurse deaths from COVID-19*.	
**Attribute**	**Execution**
Messenger	Everyday young Filipina woman from low-income family
Strategy	Describes the unique vulnerability of Filipina nurses as frontline staff, a result of structural inequalities and colonization. Shares the fears and anxieties of the daughter as she follows her nurse mothers' daily progress during COVID and share in her comfort once her mother is vaccinated.
Form	First-person narrative, mother-daughter saga
Perspective/positionality	Critical, contextual, implied directive
Setting	Naturalistic: essential workplace, home
Genre	Spoken word film
Main message	We receive the COVID vaccine as a welcome gift to protect those of us who are most exposed.

**Table 5:** Unofficial instructions for getting a COVID-19 vaccine for black girls like me, by Ciera Jevae.

https://www.youtube.com/watch?v=o_uUMo2rqeI	
This self-instructional guide offers stepwise guidance on protecting one's body at the hands of a healthcare system that has traumatized women of color. It describes Ciera's changing point of view regarding the vaccine, normatizing mistrust while ultimately accepting it. The piece highlights the undue high risk that Black girls and women take each day as they make decisions that affect their lives.	
Excerpt: *This vaccine is not a weapon against Black folks, Rather the lack of access & education is, Anti-Blackness is, Jim Crow is, Black codes, Eugenics, Capitalism, Misogyny, Every system harvesting our brilliance In an attempt to create a world by us without us*.	
**Attribute**	**Execution**
Messenger	Everyday young Black woman
Strategy	Describes the *gravitas* of decisions that Black women must make every day and frames the vaccine as an intervention that can be trusted so as to enable engagement in the larger fight against misogyny and racism.
Form	Self-instructional, self-help guide
Perspective/positionality	Critical, contextual, implied directive
Setting	Portraits in evocative scenery
Genre	Spoken word film
Main message	We receive the COVID vaccine because we must. This fortifies our resilience to enable us to combat structural violence

**Table 6:** *Me, asthmatic, immunocompromised, black, discuss coercion under the pandemic*, by D'mani Thomas.

https://www.youtube.com/watch?v=qCrOkuGw6D0	
This piece explores the exploitation of essential workers like D'mani. Unable to work in the face of COVID-19 due to his asthma and immunocompromised state, he is forced to sacrifice his income for his health. Acknowledging that vaccination in that context becomes a form of coercion, he grapples with the question of why society is willing and able to provide COVID-19 vaccines for all but lacks the will and resources to promote housing and food for all. Yet, he accepts the vaccine and looks forward to reconnecting with his people and his city.	
Excerpt: *The vaccine means I can talk to them normally. I can say, “be safe” and hug them in the same affection… How medical professionals made the Oakland Coliseum A vaccination site in mere days, But last summer we said housing for all and the city Sent military tanks to evict mothers from homes nobody was using. Last summer, wildfires made the sky bleed, and the government made prisoners patch the wound. If there was a vaccine for poverty, what would be the virus?*	
**Attribute**	**Execution**
Messenger	Everyday young Black male, essential worker
Strategy	Question the willingness of society to advance and implement biomedical solutions over socio-environmental solutions to health inequities
Form	First-person narrative
Perspective/Positionality	Critical, contextual, implied directive
Setting	Naturalistic: essential workplace, home
Genre	Spoken word film
Main message	We receive the COVID vaccine because it works but we question the long-term efficacy of such biomedical solutions in the face of structural inequalities

**Table 7:** Echo, by Yoselin Vanessa Tahay.

https://www.youtube.com/watch?v=MaN3qfMMXDE	
An undocumented Latinx immigrant describes the Herculean efforts of her large family to avoid COVID-19 infection, then deal with the inevitable household-wide infection in cramped quarters, then heal from a COVID-19-related death of a family member, and finally gain access to the vaccine–all amidst the pandemic-related economic crises that disproportionately affected low income and immigrant families.	
Excerpt: *I never imagined that in my house my entire family would be without a job And due to immigration status None of us was able to qualify for any government assistance Or maybe we were But the right resources were not provided. With a positive result in hand and bills to pay in the other We were forced to quarantine Faced with long nights of no sleep Our house echoed my father's worries, Rent was due soon and food was a comfort Listening to the cry of my mother holding my 6-year-old brother whose fever only kept getting higher, and yet he still had to attend online classes. Night after night mother was our nurse, our chef, our pillar to keep this household hopeful. Such unbearable nights not being able to hug your loved ones And slowly feeling your lungs forcing themselves to just breath, But even that was luxury, we weren't able to afford So we trusted our grandma's remedies*.	
**Attribute**	**Execution**
Messenger	Everyday young Latinx woman from low-income, undocumented family
Strategy	Describes the causes and dread consequences of COVID infection in poor families, advocates for equal access to vaccines
Form	First-person family saga, personal musings
Perspective/positionality	Critical, contextual, non-directive
Setting	Overcrowded housing interspersed with tranquil nature scenes
Genre	Spoken word film
Main message	COVID spelled disaster for low income, vulnerable communities. Equitable access to vaccines and other benefits are necessary

**Table 8:** *Should I get it, should I not?* by Mercy T. Lagaaia.

https://www.youtube.com/watch?v=Ur5mVFNVc6E	
This multi-generational piece provides an eloquent commentary on Pacific Islanders' (American Samoan's) unique risk for poor health, including COVID-19 infection. The piece, set amidst a large family cookout, frames COVID-19 as the latest manifestation of structural oppression, and receipt of the vaccine as an act to protect the entire family and preserve the older and younger generations. Excerpt: *As a people, Pacific Islanders have lacked representation in education, politics and community groups. We were born stubborn but that quickly turns into ignorance when we neglect the simple truth that COVID IS KILLING US. There were 145 new deaths reported among Pacific Islanders for the last full month data of January this year. The vaccine is meant to stop new forms of COVID. I ain't trust the vaccine at first cuz we was raised to be skeptic. I got old folks at home so I got da shot cuz it meant they'd be protected*	
*2,061 of us are known to have lost our lives to COVID through March 5th, 2022 and most of them haven't been vaccinated. We make up the majority of America's army yet they discriminate against our right to Medicaid. 90% of the reason for joining, is to give back to our families being that we come from the highest poverty rate of any state or territory in the U.S. it's mainly how we fight for a country that charges us to fix the very wounds made in their war. COVID shouldn't be the alarming evidence As to how this country always had our backs on da ropes*.	
**Attribute**	**Execution**
Messenger	Unseen young Samoan female narrator
Strategy	Positions the vulnerability of American Samoan families to COVID as a natural extension of structural discrimination, and argues for the vaccine as a means to preserve family, as represented by the grandfather and his traditional rituals
Form	Third-person narrative, family saga
Perspective/positionality	Critical, contextual, implied directive
Setting	Family picnic/barbecue
Genre	Spoken word film
Main message	American Samoans have been oppressed and colonized; the vaccine is a ticket to survival

**Table 9:** *Virus*, by Jamaia Lincoln.

https://www.youtube.com/watch?v=MdUjtynkKR0	
In this first-person testimonial, a young Black, newly matriculated college student describes how COVID-19 disrupted university life, and how she is making an exception regarding her usual mistrust in government by trusting this “priceless” vaccine. While acknowledging the myriad structural drivers of COVID-19 deaths, she celebrates the vaccine as an option to choose life and to enable the experience of our rites of passage.	
Excerpt: *I could tell you Black folk have died the most from the virus, that 20% of people of color in the U.S. didn't have enough bedrooms or bathrooms to even quarantine properly. That the system has been designed to increase our disparities–to see us unalive. I could tell you nearly all the ingredients in COVID-19 vaccines are also ingredients in our food—fats, sugars, and salts. I could tell you people who get the vaccine have a 95% lower chance of getting sick of lying with their parents, friends, cousins, dead body Or I could tell you, that my grandmother cried when I got off stage and handed her my diploma. That my uncle's mother survived COVID after her husband's death. that mothers can find space in more than the bathtub. that children are sharing public playgrounds, and students are learning goldfish for the first time in their dorms. We have the option to choose community, to choose life, together, beside one another, It's priceless*.	
**Attribute**	**Execution**
Messenger	Everyday young Black female college student
Strategy	Describes how health inequities are driven by structural societal drivers but acknowledges that surviving to be able to enjoy rites of passage requires a form of exceptional acceptance of the COVID vaccine.
Form	First-person narrative, family saga
Perspective/positionality	Critical, contextual, implied directive
Setting	Naturalistic: college dorms and dining halls, home
Genre	Spoken word film
Main message	We should accept receive the COVID vaccine if we want to enjoy life's rites of passage

**Table 10:** *Oak Wilt*, by Huey Colette.

https://www.youtube.com/watch?v=0aHkr0WcR3c	
Framed as an intellectual conversation between two friends in Stockton, CA, the poem reflects on how society views homelessness—its causes and consequences—as an individual rather than a societal failure, and draws parallels with how COVID-19-related disparities are viewed. In calling out that change is needed to move away from individualism and toward a communal response to inequity, the poet acknowledges that a vaccine prepared by those in power—administered one individual at a time – can allow us to return to a sense of community.	
Excerpt: *The vaccine being a trade off to the marginalized. A willingness to forgive atrocities, experiments, and a profit for protection against the virus. But to breathe again, See the vibrant souls of my town pulsate through murals on the wall, While shelters implement vital stratagems to reach the homeless. “95 percent efficacy, 100 percent effective in preventing serious illness and death” And though immunity from Covid-19 is only the initial shot for my people from Oakland to Stockton… A vaccinated future contains the power to reconnect, While enabling the unification of raised fists against the establishment, To brush off these societal cobwebs we find ourselves in, Ripping the generational adhesives between the ill-fitted labels*.	
**Attribute**	**Execution**
Messenger	Every day, philosophizing young Black man
Strategy	Positions the public discourse around COVID disparities as dysfunctional in its focus on individualism
Form	Heady conversation between two friends
Perspective/positionality	Critical, contextual, implied directive
Setting	A drive around the Central Valley
Genre	Spoken word film
Main message	We can accept the COVID vaccine, one individual at a time, because it will restore our sense of community.

### Evaluation and analysis

We conducted this evaluation study in two phases. In phase 1, we examined the campaign materials to explore the myriad ways that young poets of color participating in *Survival Pending Revolution* created novel content that is closely aligned with many of the attributes and objectives of critical communication theory. Specifically, we subjected the campaign materials to a thematic analysis whose purpose was to identify the range of themes related to framing and argument-making within and across the nine poems, as well as to quantify which themes were most prevalent, assessing the extent to which this campaign reflects a critical communication approach. In Phase 2, we conducted a focus group with members of the target audience (YOC) whose purpose was to elicit reactions to, and opinions of, the content presented in the *Survival Pending Revolution* campaign relative to content from the Kaiser Family Foundation's well-established and widely viewed COVID-19 vaccination/equity campaign, *The Conversation*. This campaign was chosen as a comparator because it is considered to be of high value for minoritized populations; it was created by a highly regarded health communications entity with the support of numerous respected stakeholders; it was specifically tailored for marginalized subgroups; and it exhibits what many consider as best practices in vaccination communication for minoritized groups. (12) We then carried out a thematic analysis to identify and compare the campaign-specific themes that arose from this focus group. Our overall purpose was to provide preliminary data to the California Department of Public Health about the potential efficacy of the *Survival Pending Revolution* campaign to inform future dissemination efforts. Insofar, as the research we present references the campaign content, we encourage the reader, as they progress through the analyses in this report, to view the brief video-poems *via* the links provided.

#### Phase 1 (poetry-based data analysis)

We have previously utilized qualitative analysis to uncover the extent to which the socioecological model of health was communicated in an arts-based diabetes prevention campaign (8). We adapted this approach to analyze the nine video-poems from the *Survival Pending Revolution*. First, two coders—one a spoken word poet and the other a public health communication specialist—separately viewed each video-poem and then reviewed the written transcripts of each poem using text files. Using a consensus process, the coders, using a directed analysis approach, then characterized the attributes of each poem across the domains listed in Table 1 (above) as a preliminary qualitative measure of alignment with critical communication theory. Second, the two coders, again using a consensus process, coded each poem for (1) arguments relating to the rationale presented for COVID-19 vaccination and (2) contextual contributors of COVID-19-related suffering and inequities presented. Using reflexive thematic analysis, (13) we aggregated individual codes into overarching themes.

#### Phase 2 (focus group)

On 1 September 2022, our investigative team carried out a 90-min, online focus group involving a convenience sample largely representative of the intended target audience. Invitations to participate in the focus group were sent by a Program Director at *Youth Speaks via* email messages and social media postings 1 week prior to the event. Outreach emails were sent to all local partners, including past and present partner schools, libraries, allied individuals, and community-based organizations. Two video invites were issued on social media *via* the Instagram and Facebook “stories” function and sent to *Youth Speaks*' network. Individuals residing in California, aged 18–30 years, were eligible to participate. Participants were offered $50 to engage in the focus group.

Our project evaluation budget allowed for participation of up to 15 participants. Anticipating that some respondents to the invitation might not show up for the focus group, we aimed to recruit 18 participants. Because the content that was to be tested targeted Black and Latinx audiences, we intended that at least half of the participants should self-identify as Black or Latinx. Because we were also interested in the perspectives of other audiences of color, we did not limit recruitment to these two racial and ethnic groups. We did not specify any requirements regarding participant gender. Once the first 18 individuals affirmatively responded to our invitation, we closed the invitation.

The focus group was facilitated by an experienced Program Director at *Youth Speaks* (Gabriel Cortez). In addition, the evaluator (Dean Schillinger MD, a public health physician and an expert in health communication) was present to introduce the purpose of the focus group and to serve as an observer. With the consent of all participants, the focus group was videotaped; the closed captioning function was activated to enable live transcription.

### Comparative content presented

During the focus group, participants were first exposed to a set of videos targeting Black audiences (one video from *The Conversation* and one from *Survival Pending Revolution*). They subsequently were exposed to a second set of videos targeting Latinx audiences (one video from *La Conversacion* and one from *Survival Pending Revolution*).

In the first set of videos, participants were shown the following:

1. A complete segment from *The Conversation* campaign that targets Black audiences (duration 3 min 27 s). It is entitled: “W. Kamau Bell Talks with Doctors About Why You Shouldn't Wait to Get a COVID Vaccine” and can be viewed here:

https://www.youtube.com/watch?v=AnyJSC3PYM8

This content features the Black television celebrity, W Kamau Bell, in conversation with actual Black healthcare providers (primarily physicians in white coats). The video has a lighthearted question-and-answer-type format, with Bell asking questions that reflect the concerns about vaccination believed to be commonly held by the target audience, and physicians providing clear and reassuring responses whose purpose is to build a sense of confidence and urgency regarding vaccination. At the time of our focus group, this video had been viewed on YouTube by over 266,000 people.

2. A complete video-poem from the *Survival Pending Revolution* campaign (duration 6 min 19 s). The poem, entitled “A Walk Through the Valley” was written and performed by Nia Lundkvist, a 24-year-old Black artist from Oakland, CA, and can be viewed here:

https://www.youtube.com/watch?v=fzOmA40Vge0&t=1s

The format of this piece is first-person narrative: it tells the story of Nia's conflicting thought processes and ambivalent emotional journey leading up to her COVID-19 vaccine. In the telling of this story, she both articulates the compelling need for protection against COVID-19 in her community due to structural racism, while also enumerating how medical racism and mistrust undermine her faith in the vaccine, making her decision-making process both complex and harrowing. At the time of our focus group, this video had been viewed on YouTube by 1,860 people.

In the second set of videos, participants were shown the following:

1. A complete segment from the *La Conversacion* campaign that targets Latinx audiences (duration 3 min 53 s). It is entitled “Why the COVID-19 Vaccines Matter For Us” and can be viewed here:

https://www.youtube.com/watch?v=PwQULDbQ5ac&list=PLA9jKZBtI0RI-TThvQaDYsEv3cwRX3cCT&index=1.

This content features Latinx healthcare providers (physicians in white coats, nurses, and community health workers) describing their real-life emotional experiences of relief, gratitude, and joy upon receiving the first COVID-19 vaccine. The video includes a focus on the role of vaccines in enabling the re-establishment of connection to family and community. At the time of our focus group, this video had been viewed on YouTube by over 229,000 people.

2. A complete video-poem from the *Survival Pending Revolution* campaign (duration 4 min 26 s). The poem, entitled “Cuantificando Lo Esencial (Quantifying the Essential)” was written and performed in Spanish (with English subtitles) by Sandra Vazquez, a 21-year-old Latinx artist from Richmond, CA, and can be viewed here:

https://www.youtube.com/watch?v=lHMTWXCJ6KA&t=1s

The format of this piece is also first-person narrative: Sandy tells the story of her mother and aunt, both essential (likely undocumented) workers who are continually exposed to COVID-19 as a result of their jobs. In this piece, she acknowledges the unique vulnerability of her family and community with respect to COVID-19 and a host of other unhealthy exposures, while also celebrating the resilience of her family and community to survive. Her poem includes an open acceptance of the COVID-19 vaccine while also calling out the need for larger, systemic solutions that go beyond a vaccine. At the time of our focus group, this video had been viewed on YouTube by 753 people.

After each set of videos was presented, the facilitator invited a compare/contrast conversation in response to the following prompts:

- What do you all think about their content and their presentation formats?
- What do you think the main message is?
- What spoke to you/resonated with you about this video? What did not?
- What did you think about the fact that medical “experts”/regular people who represent certain communities are the featured speakers who convey facts and info to you?
- What did you think about the emotional tone?
- What did you think about its entertainment/engagement value?
- Did you feel this video provided credible information (info you could trust)?
- Regarding COVID-19 vaccines, did you feel that the video is asking you, or people like you, to do anything? Are you OK with that degree of direction?
- In summary, how effective do you think this video is for you and for people in your community?

#### Phase 2 (focus group analysis)

Two weeks after the focus group, the same two coders who carried out Phase 1 separately reviewed the written transcript of the focus group, employing reflexive thematic analysis (13). First, they created a set of codes that reflected and were linked to each participant's response to the open-ended prompts associated with each set of videos. Coders started by reading all data repeatedly to achieve immersion and obtain a sense of the whole. Then, data were read word-by-word to derive codes, highlighting the exact words from the text that appeared to capture key thoughts or concepts. Coders made notes of their first impressions, thoughts, and initial analysis and labels for codes emerged that were reflective of more than one key thought. These codes were then sorted into categories based on how different codes were related and linked, with emergent categories organized and grouped into meaningful, overarching themes.

## Results

### Phase 1

In Tables 2–10, we characterize the attributes of critical communication theory present in these video-poems. As is evident, nearly all poems aligned with principles of critical communication theory across multiple domains, contrasting with the attributes of more traditional public health communication content. In all cases, the *messenger* represented regular, everyday people who reflected the target audience. The dominant *strategy* employed, rather than focusing on fact-giving or fear-inducing, tended toward a calling out of societal and structural injustice as a critical context for disease, as well as some form of a call to arms to overcome power imbalance to achieve health and wellbeing. The *form* of most pieces featured a narrative accompanied by compelling emotional content and tone, often involving family relations. The *positionality* of nearly all pieces was non-authoritative, tending toward a critical perspective that uses a bottom-up approach to question the dominant power structures and “party line”. Similarly, the *setting* for each piece was unexceptional, reflecting the everyday, lived experiences of the messenger. Finally, the *genre* was inherently artistic, avoiding the risk of being perceived as instructional or didactic.

#### Dominant themes

**Figure 1:**
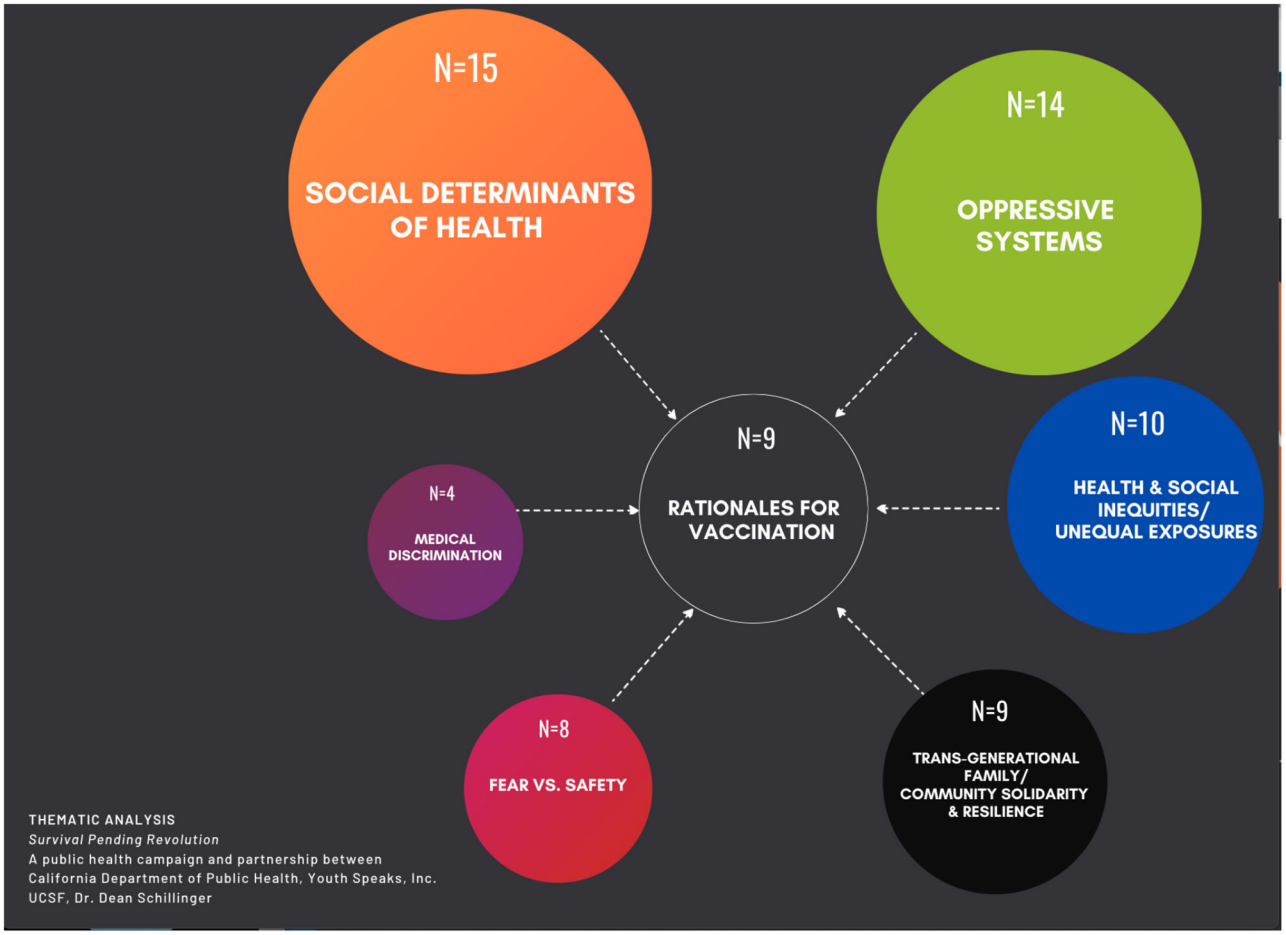
Major themes in video-poems (*N* = 9) of *survival pending revolution* campaign.

We then carried out content analyses whose purpose was to identify the range of themes within and across these nine poems, as well as to quantify which themes were most prevalent. These themes demonstrate the ubiquity of critical inquiry and the depth of critical analysis as conveyed by the artists in their pieces. As displayed in the center of Figure 1, each of the nine poems summarized in Tables 2–10 communicated a rationale for COVID-19 vaccination. These included the need to survive to fight a larger battle for social justice (*N* = 3); a desire to feel safe/protected (*N* = 2); return to a sense of community/ability to allow us to live and to celebrate life (*N* = 2); the need to preserve a way of life (*N* = 1); and accepting the vaccine as a necessary compromise/trade-off (*N* = 1). The contextual content surrounding these rationales fell into six overarching themes, which we present in descending order of prevalence. The first theme was Social Determinants of Health (*N* = 15) and included poverty (*N* = 4), employment/essential worker status (*N* = 4), housing insecurity (*N* = 3), and immigration (*N* = 4). The second theme was Oppressive Systems/Structural Determinants (*N* = 14) and included structural violence (*N* = 6), capitalism (*N* = 4), colonialism (*N* = 2), individualism (*N* = 1), and misogyny (*N* = 1). The third theme was (3) Health and Social Inequities (*N* = 10) and included unequal exposure to risks (*N* = 6), health inequities (*N* = 2), and social inequities (*N* = 2). The fourth theme was Trans-Generational Solidarity and Resilience (*N* = 9) and included family (*N* = 6) and community (*N* = 3) levels. The fifth theme was Medical Discrimination (*N* = 4) and included medical racism (*N* = 2) and unequal/discriminatory access to care (*N* = 2). The sixth and final theme was (6) Mistrust of Medicine and Government (*N* = 3).

### Phase 2 (comparative focus group)

Thirteen individuals attended the online focus group in its entirety. The mean age of participants was 25.5 years (range 18–34). Three identified as Black/non-Hispanic, one as Black/Latinx, four as White/Latinx, four as Asian/Pacific Islander, and one as First Nations/Indigenous. Ten were identified as women and three were identified as gender non-conforming. All participants were actively engaged throughout the focus group, and each provided one or more responses to the prompts involving each set of videos. As mentioned later, we identify the major themes that emerged in response to the video viewings and prompts. We also include representative quotes from participants. We denote quotes that reference *The Conversation* with “TC” and quotes that reference *Survival Pending Revolution* with “SPR”.

### Theme 1: validating complexity in vaccine decision-making

Perhaps the most predominant theme that emerged when participants compared the approaches used in each campaign relates to the perceived oversimplification of the problem and its solutions when presented in *The Conversation* vs. the validation of complexity when presented in *Survival Pending Revolution*. When reflecting on *The Conversation*, participants frequently described how turned off they felt by the relatively superficial and even disrespectful analysis of the decisional uncertainty related to COVID-19 vaccination faced by marginalized populations. One participant reported that she felt that this approach was insensitive and represented a cancellation of valid concerns:

(TC) *I think the first video is very dramatized. And I don't know if insensitive is the right word or like, I don't know, just like very superficial. There was just like an overemphasis on like both ends… like from the doctors who were speaking…and then the person who was supposed to be like bringing up the questions that are like actual concerns that people have was like asking them in a very flippant way…Like that's an actual concern that he had, but it was more like “This is like a crazy question that people bring up when they don't wanna get the COVID vaccine.” And like, now these doctors are gonna answer it and like, almost in like a demeaning way*.

Another participant concurred feeling that convenient shortcuts were being made that sidestepped authentic worries:

(TC) *But I definitely do feel like it was a little bit flippant the way the questions were being asked. And even though they were real concerns that you hear people saying. It seemed like it was like tick, tick, tick, tick, tick, tick. Like, let's just check off all the boxes to where people don't have an excuse to not get this vaccine. And it seemed that like at the end, the last sentence was “The vaccine is the only way we're gonna get out of this…And then this last thing that they said was like “And it's the only way.” And it's like, what? Like we didn't get there together.”*

Many participants specifically called out how the tone and content of *The Conversation* assumed that people of color have comfort with the healthcare system, ignoring how they have been traumatized by the healthcare system:

(TC) *It seemed like they probably wanted to bring in some lightheartedness to a very serious situation. I don't think they did it in a very good way. And then just also kind of felt off put by the fact that they didn't address the fact that people have inherent trauma around the healthcare system*.

Many participants reported that they felt that the lighthearted tone of some of the content in *The Conversation* was inappropriate, almost tone-deaf.

(TC) *Yeah, definitely feel like there was a huge contrast. I think in the first video the first person who started talking was kind of addressing their concerns around being vaxed or not, but it was, I felt like it was portrayed very like in a comical way. Right? Like the goofy vibe or like very, I don't know, it was just, it was different in regards to a topic that can be very serious*.

In contrast, most participants seemed to appreciate the ways in which the *Survival Pending Revolution* campaign took POC's concerns seriously, illuminating the complexities that underlie these concerns and that make decision-making for them less than straightforward. One participant expressed her appreciation for this approach as follows:

(SPR) *I think for the second video, there was just so much more context to like something that might seem very binary, right? Like you're either getting vaxed or not, but it's just so much more than that. And so I appreciated that being expressed in the second video. And the second video, I think did a better job, especially in the first half, of validating people's like actual concerns around the healthcare system*.

Another participant described how the poet's description of her ambivalence with respect to trusting the doctor to vaccinate her resonated with the participant's own sense of mistrust of physicians:

(SPR) *So I just noticed that like, doctors are always portrayed as like, they know, they know what, like they're gonna take care of you. Trust them. Like, I don't trust my doctor all the time. So, like, not just around COVID. Just like my doctor, my primary doctor, isn't Black. So I'm like, yeah, those are valid concerns to have as a person of color in general. I think I already said it, but yeah, doctors don't know much about every race, especially Black people. So, I'm like, it's kind of hard to trust them all the time…I think the second video did a good job at highlighting that like, I don't know another word, but like mistrust or even hesitation to trust your doctor*.

Another participant echoed the sentiment as follows:

(SPR) *The second video though I think it did like a much better job of like honoring people's anxieties about the vaccine…I did feel validated when she was looking at the doctor, like, like, I think, I, I don't know if it was ambivalence, but, or more or more like “I need to make this decision for like other people.” And like, yeah, I don't, I don't know if I picked up ambivalence, but…I did appreciate the, like the, seeing the doctor, because that's how it feels, you know, like people are just like, ding, ding, like you wait in line to get your stuff. You're like, ding, ding. And they're just like, OK, bye. Like without no disregard for what you had to do that day*.

### Theme 2: harnessing authentic vs. inauthentic emotion

Participants reflecting on the approach used in the *Survival Pending Revolution* seemed to value the poet's ability to communicate the authentic emotional struggles she was going through as she was preparing to get vaccinated. One participant commented that the conveying of emotions in these pieces both humanized and promoted retention of the messages:

(SPR) *I retained so much more information with the poem. It was able to walk me through all of these feelings and emotions that came up when I was watching the other video. So it humanized the situation. It was relatable*.

Another participant felt that the display of emotion made the spokesperson a more effective advocate for COVID-19 vaccination:

(SPR) *And then for the second video, I really appreciate how, you know, how she reveals all her personal feelings and all the details. And, you know, and I think to me, like that means a lot…The second video [made her] come off as more like a, you know, like an advocate*.

For another participant, the poet's use of the first-person narrative form brought out the emotions and the details in a way that made her message more real and more powerful:

(SPR) *For me the second one was more powerful and it definitely like pulls at my heart strings. I thought it was a powerful narrative through both poetry and images. And I loved the specificity of the narrator's story. Like, you know, hearing the specific $20 Ride Share really hit…I felt like that really like captured a genuine experience. That's hard to capture just with words*.

In contrast, participants reflecting on *The Conversation* reported uncertainty regarding the emotional authenticity of the spokespeople. This uncertainty undermined the credibility of the messenger. One participant stated:

(TC) *So, the first video I felt kind of uncomfortable that this, you know, that man was so excited. And it made me like, kind of feel like I was questioning him, like, is he trying to trick me?*

Another participant felt that the emotions conveyed by health professionals in *The Conversation* did not resonate with her own experience and were not relevant to her:

(TC) *I understand like they have their own reactions, like when they were crying and dancing and so celebratory. I understand that as doctors that might be their reactions or a few of their reactions, but I, I, that was not my reaction to getting the vaccine nor was it any of my homies. So, I found that part of the segment kind of just a little bit off*.

Another reported that the absence of a personal narrative in *The Conversation* undermined the authenticity of the spokesperson:

(TC) *The first video, like the man that was like, kind of like questioning and asking questions. I felt like that would have been like, you know, like, I think if it's like a more serious phase, like with like some like concerns… I think looking at his emotion, I didn't feel like it was very personable. Like it was, it wasn't really about his personal experience*.

### Theme 3: historical and racialized context matters

The absence of contextual content related to the underlying drivers of health inequities was perceived as a notable deficit in the content of *The Conversation* campaign. One participant felt that this blind spot meant that the content was nothing more than:

(TC)…*a bunch of medical professionals spouting information that you'll hear or see kind of just on different media platforms*.

Another participant felt that the campaign's lack of acknowledgment of the sociopolitical context, that drove the disproportionate suffering related to the pandemic, undermined the credibility of the campaign's COVID-19 vaccine message:

(TC) *Like in the first video, they're like, “Oh, we don't have to be scared anymore!” It's like, where? Like, where's what, like, that's the only…well, like, that's just thing one!*

In contrast, participants reported a greater openness to the messages of the *Survival Pending Revolution* campaign as a result of the inclusion of sociopolitical context, including mentions of structural racism and the structural determinants of health. One participant reported:

(SPR) *And it really discussed, not only COVID and health, but so many underlying issues that come when getting the vaccine because of all these different systems in place. The second video really resonated with me just with the more context that was given. And then I loved how it expanded into like larger systemic issues like the Tuskegee references and…It felt like it was kind of just showing like this is how we got here*.

Another participant appreciated the focus on what comes after the vaccine, on how an understanding of context leads one to an understanding of the limits of vaccination as a temporary solution, not the ultimate solution:

(SPR) *That's what I really appreciated about Sandy's video. That even after they got the vaccine there was still like distrust in the government and emphasis on it as a temporary solution. And I felt like that was incredibly important to have in there. And I also really appreciated how they kind of answered implied questions or questions that you don't necessarily see in some of those informational-based videos*.

A related perspective on the importance of framing the vaccine decision within the larger context of social inequality was made by another participant:

(SPR) *I think, you know, I think it really did a good job of showing different phases and like the living situation of a vulnerable population. Like one of the example of like, you know, a family who have to work in stores…I think like one thing though, like a limitation would be that, you know, like…many marginalized and underserved groups are really traumatized. And, you know, they already facing so much like structural inequalities and like, you know, getting vaccine may not necessarily like relieve them*.

### Theme 4: questionable credibility and actionability

In response to *Survival Pending Revolution*, there was no consensus around whether the spokespeople were felt to be credible. In some instances, participants felt that the naturalistic style of the poems narrowed social distance, allowing participants to relate to the poets as people just like them and to relate to their messages:

(SPR) *I frankly really appreciated that the poet was a black female presenting... And it was very nice to kind of hear from somebody who looks like me. Being on the front lines and getting sick, but also feeling so unsafe on how we're being treated in the health system*.

In other instances, participants questioned the intent of the poet and felt that the poet, and the poetry, were being appropriated by those in power to do their bidding—to convince people of color to get vaccinated to protect everyone. For some, this perception undermined the credibility of the messenger.

(SPR) *Even though I super appreciate the poetry, like my first reaction was like, oh, here, they're trying to, they're trying to get the Black folks and the Latino folks, like with the poems, like here we go, you know? And its' Youth Speaks so, you know, we can expect the poems, which is amazing. And I love them, but I almost got this feeling of like, oh God, you know…But now that we're trying to get the Black people to do something like we're gonna get a poem going. And that feels, it feels in line with the message that she was saying, right? Like, all around us, like people don't care. But now that they want us, they need us to do something. Like it's very much like, you know, like disingenuous almost*.

This sense of Black and Latinx art and culture being co-opted undermined some participants' ability to accept the messages related to COVID-19 vaccination and the logic of the poets' journeys. One participant described this disconnect and experienced it as a sign of manipulation:

(SPR) *I'd really like to hear about like what the doctors are doing and what has been done by them to, like, gain our trust back. Like, what are you doing? Like what's happening because also at the end it was also very stark because she went from, these are all the terrible reasons I don't trust the government to like, “Oh, I'm gonna get this shot for, for me and for the home”, you know? And it was like, well, how did we, did we get any of our questions asked? Like, are there any provisions? Is there a plan for us? Like what is gonna happen? And it like boils down to individual responsibility. Whereas the systems around us and the institution around us are, okay, failing us. And so it's it, it like I appreciate, I appreciate the information, but I'm kind of, there seems to be jumps. And, and within those jumps, are gaps that seem clingy and, and disingenuous. Like you can tell that they're that they're trying to get us to do something*.

Another participant felt a disconnect between the acknowledgment of the larger systemic problems and the presentation of an individually based solution:

(SPR) *We're having to tell our story over and over and over again for somebody to be like, “Please listen, like, please listen”. And then it's like…we're showing it to try to convince us to do a thing. Like we have to do it ourselves*.

One of the strategies of the *Survival Pending Revolution* was to give voice to the tensions between the need to get vaccinated and the anxiety related to medical mistrust as well as the need to simultaneously transform our society to promote health equity. While this strategy worked for some participants, it was not always effective in promoting behavior change for others:

(SPR) *I don't feel like I would have left that video being able to make an informed decision on, like whether I wanted to take, to have the vaccine or not*.

In addition, while some participants valued the use of art as a medium to convey health messages, one participant raised concerns about this strategy:

(SPR) *It's a poem. So, we kind of had to like listen to what she said and maybe like, go back and like, oh, I missed the line. Like, what does she mean by that? Or like watch the video to understand what she was talking about*.

In contrast, this participant felt that the more direct line of instruction prevalent in *The Conversation* was less demanding and more helpful to her:

(TC) *You could hear video number one and like, not even look at the video, just to like, okay, they want me to go get the vaccine or at least talk about it. Like you were saying, like that can spark any type of conversation*.

Another participant felt that the clear communication style of the physicians in *The Conversation* and its more traditional style made it credible:

(TC) *The three female doctors who were, you know, like sitting like really in a formal setting and, you know, it's more like the set up the camera and everything. It's really like, you know, like, like a formal kind of filming, whatever. Like, I don't know that word, but …I think that gave me like…kind of like helped establish more like credit. Like for me, to trust, to like the video. I think I also really like the terminology, like kind of the registry of the vocabulary. Like it wasn't like really like medical terminology. It's more like…daily words and, you know, the logic makes sense to me. So, so I felt like it was very convincing, like from the doctors*.

As with *Survival Pending Revolution*, the effectiveness of *The Conversation* was highly variable and was perceived to be generally ineffective at changing behavior:

(TC) *The presenter was approachable, and the doctors were reputable, I suppose. But it felt like it was more for people who are already like pro-vaccines and want to convince other people with it. So, it would be like a resource for them to turn to. I think that if I were on the fence, I'm not sure that it would play for me*.

As described earlier, some reported that the approach and logic in *The Conversation* were simplistic. They also noted that the overly optimistic messaging further undermined credibility:

(TC) *So like, if you wanna see your family, you have to get the vaccine. So, I feel like it was kind of like, oh, there they're going out now. Like, because they got the vaccine. So, I feel like it was another video to kind of persuade people to do so. Like they were making it sound like now we can go out and see family. Like, no, we barely just started like doing family gatherings again barely last year. And it's been like 2 years, almost 3 years since COVID started. So, I'm like, hmmm, I don't know. It was just a hesitation toward that video for me*.

## Discussion

Critical approaches to health communication presume hegemonic structures and, importantly, they speak to the need to rectify concomitant social inequities and injustices that impact an individual's lived experiences of health and wellbeing. Such approaches to health communication recognize that certain sectors of the population have been marginalized by institutional practices of policymakers, interventionists, and program planners and that this marginalization increases the risk of disease. The goal of critical communication is to aid such populations in both resisting and navigating a system that continues to locate them on the margins of society. It is always a challenge to discuss the implications of paradigmatic differences without seeming to take an “us vs. them” attitude. Can public health appear to embrace such an antagonistic stance toward authority and the establishment?

The content analysis of the video-poems presented in Phase 1 of our evaluation suggests that the young artists in this campaign answer this compelling question with a resounding “yes”. They appear to recognize that, for their communities, the only path to productive dialogue lies in acknowledging the sources of our tensions, recognizing the contributions of alternative perspectives, and negotiating acceptable compromises. Many of these poems give voice to these dichotomies; they give expression to how these young people navigate conflict—at times through resistance, at times through compromise, and at times through faith. But always doing so from a place of power and agency, and with a perspective of the long view. In one way or another, they all communicate to their audiences that COVID-19 is but one battle in a larger war against structural violence and that getting the vaccine provides an important path to survive for that larger fight.

We conclude that engaging YOC to develop artistic content as a means to engage their peers to consider COVID-19 vaccination *via* a collaborative and participatory approach facilitated by public health practitioners and artists was an effective method for the creation of critical communication content by this cohort of youth. Enabling YOC to view their “life as primary text” led them to produce art that conveys the complex, multilevel mechanisms of disease and health disparities—in novel, compelling, and relatable ways (14, 15). The art also provides authentic narratives that demonstrate personal and communal agency in navigating critical public health decisions in the context of historical and extant structural violence.

Poetry and other forms of expressive writing have previously been identified as a therapeutic tool to both foster self-control and voice prior trauma while accessing one's unconscious self (16), and have been described as effective in dismantling oppression for social change *via* group-centered leadership and collective transformation (17). While there remains a paucity of quantitative data to support the impact of poetry on social change, extensive narrative history dating back to the origin of the Greek word *poiesis* links poetry to the formation of ideas that communicates societal creativity able to transcend from individuals toward society at large (18). Inherent in its origins, social change requires improvisation and collaboration built on old traditions; as such, poetry informs praxis as the underlying dialogue between the arts and human activity. Youth—skilled in both improvisation and collaboration—can be powerful catalysts for public policy change, exemplified by the efforts of Floridian youth leaders and their #NeverAgain social-media campaign against gun violence (19).

Poetry provides a fertile canvas for the exploration of identity and environment, inviting the opportunity to question historical knowledge within predefined socioecological determinants. There is a body of evidence to support harnessing spoken-word poetry and hip-hop culture both in youth-based programming as well as in cognitive behavioral therapy as a means to contribute to better psychological wellbeing (20–22). Spoken-word writing in youth-focused programming has demonstrated validity in empowering critical thinking that allows some youth to achieve positive emotion, engagement, relationships, meaning and accomplishment—the core building blocks of wellbeing and self-expression that may propel behavioral change and community connectedness.

In Phase 2 of our evaluation, we carried out an exploratory qualitative study to assess the efficacy of the spoken word content presented from the *Survival Pending Revolution* campaign relative to the content presented from the Kaiser Family Foundation's COVID-19 vaccination/equity campaign, *The Conversation*. To the best of our knowledge, this is the first study to compare the perceived effectiveness of an arts-based public health communication campaign, whose design was aligned with critical communication principles (see Table 1), with a more traditional, state-of-the-art communication campaign. The focus group involved 13 YOC, with over half of these individuals representing the race and ethnicity of the intended target audiences. Overall, participants viewed the content from the novel *Survival Pending Revolution* campaign in a significantly more favorable light relative to the more traditional *The Conversation* campaign across several dimensions of engagement, including emotional resonance, authenticity, and validation of lived experiences. However, neither campaign appeared to generate consistently high degrees of credibility or behavioral activation or intent. The results of this focus group provide insights into the potential value of critical communication approaches to public health communication and provide some evidence in support of specific attributes of the novel *Survival Pending Revolution* campaign. These insights open up a broad range of possibilities for constructively disrupting traditional public health communications and could provide novel strategies to combat mis- and dis-information (23).

First, positioning a young poet of color from their own community to serve as the spokesperson, or messenger, of their own public health content appears to have several advantages over the more traditional approach (using celebrity or healthcare provider spokespersons). These advantages included greater degrees of authenticity, higher relatability, and greater perceived concordance with the lived experience of the intended audience. This appeared to create a communication context for participants to be more open to hearing the campaign's messages. Second, the narrative, naturalistic, “bottom-up” form of *Survival Pending Revolution* notably contrasted with the more didactic, informational, and “top-down” form of *The Conversation*. This storytelling form appeared to enable the sharing of emotionally compelling content with the audience, and this emotionally compelling storytelling was experienced as enabling greater engagement with and retention of health information. Third, the open acknowledgment of the multi-level and complex structural determinants of health present in *Survival Pending Revolution*—for example, the calling out of structural racism as a real issue that needs to be contended with as one considers how to respond to the COVID-19 crisis—was experienced by all participants as superior to the more simplistic, individual-level solutions proposed in *The Conversation*. The validation of the effects of medical and political trauma on the lived experience of POC appears to have provided the needed buy-in for engagement with, as well as a welcome degree of gravitas, the *Survival Pending Revolution* that *The Conversation* did not enjoy. Fourth, the use of art as a means to communicate health messages appeared to have succeeded not only in enabling the expression of emotion but also in conveying the contextual complexities that make such health decisions challenging for marginalized communities. However, for a few participants, the art—while very engaging—was less effective in conveying information relative to the more straightforward fact-giving. For others, the use of a culturally relevant art form was perceived as an appropriative act that undermined credibility. Finally, the non-directive nature of the critical communication approach apparent in *Survival Pending Revolution* appeared to be no less effective in influencing behavioral intent than the more traditional and directive approach evident in *The Conversation*. Importantly, we found no evidence that this non-directive approach engendered behavioral intentions that were in opposition to vaccination.

It is important to situate our study within the existing knowledge base related to vaccination campaigns and critical health communication more broadly. Recent work related to understanding and increasing COVID-19 vaccination rates has tended not to focus on the concerns of POC. Most research has involved older adults and has focused on the influence of political ideology on COVID-19 vaccine acceptance and uptake (24). A large survey of young adults confirmed that about one-quarter of respondents reported no intention to get vaccinated, with the most common reasons for rejecting being concerns about safety and side effects, and belief that others need it more (25). However, fewer than 8% of respondents were Black. While recent experimental research has attempted to determine how to overcome mistrust of vaccines, most interventions have involved different designs of messages delivered by experts conveying information based on their expertise, (26) including scientific messages related to vaccine safety and side effects (27). While a recent review of strategies to promote COVID-19 vaccine uptake among minoritized populations recommended addressing mistrust and misinformation by considering message content, messengers, and setting, such studies have focused on messages coming from Black physicians, faith leaders, and barbershops (12), and have not considered whether they meet the needs of YOC. Vaccination communications intended to highlight that the dangers of disease run the risk of leading to increases in beliefs about the harms of vaccination—the opposite of the expected outcome. Nyhan et al. conclude that “current public health communications about vaccines may not be effective” (28). Most public health approaches to vaccine promotion remain rooted in theories of change, many of which were developed in the 1950s and 1960s. It is too often assumed that people are vaccine-hesitant because they do not have the correct information. However, health communication research suggests that knowledge, while important, is not enough to change behavior (29). Central theories of health communication campaigns that represent the individually focused, cognitive approach (theory of reasoned action, health belief model, and the parallel process model) often ignore historical and social contexts and may be ineffective for marginalized populations (30). More recent scholarship has advocated for a critical praxis, one that centers the voices of the marginalized in ways that challenge and reshape the multi-level structures that determine health. Along these same lines, scholars have argued for the integration of the principles of socioecology and critical pedagogy for health promotion and health literacy interventions (31). and have called for a critical approach to science communication that highlights inclusion, equity, and intersectionality (32).

Despite the chorus of voices advocating for health communications to embrace a critical communication approach, little empiric research has been published that describes such campaigns, (33) and even less has been published that reports on their evaluation (34). This paucity of research is especially notable in the context of COVID-19, given the disproportionate burden that the pandemic has placed on marginalized populations (35, 36). Our study attempts to begin to fill these gaps, delivering insights into the fundamental ways that a critical communication approach can influence multiple attributes of a campaign, as well as providing a preliminary assessment of the likelihood of success of such a campaign for its intended audiences.

### Limitations

This two-phase qualitative evaluation has several limitations. Our Phase 1 study, which relied on a qualitative analysis of poetic content, while innovative and informative, may suffer from threats to internal validity, a problem related to any attempt to objectify poetic inquiry (21). Analyzing written words, particularly those extracted from performative art, is potentially fraught, limited by the dependability and confirmability due to coders' unique relationships with words or phrases, and their varied lived experiences. We attempted to enhance confirmability by including one poet coder and one non-poet coder and by using a consensus process when making coding decisions.

The design of our Phase 2 study (focus group) does not lend itself to making definitive conclusions regarding the efficacy of a critical approach to public health communication relative to the more traditional approach with respect to promoting the uptake of COVID-19 vaccination. First, this was a small-scale, qualitative study whose purpose was to explore whether there are perceived advantages and disadvantages to this novel approach from the perspective of the intended audiences. Quantitative studies with much larger sample sizes and explicit, measurable outcomes—including behavioral intentions and actual receipt of the COVID−19 vaccine—would be needed to make definitive conclusions regarding efficacy. Second, while our study suggested that the strategies used in *Survival Pending Revolution* had distinct advantages, these findings were obtained in an *in vitro* setting: participants were required to watch the specific video content from both campaigns in their entirety. It is possible that a more naturalistic, *in vivo* study, in which participants could watch as little or as much of the content as they wanted, would generate different results. Third, as with most qualitative studies, the small sample size and the sampling strategy we used may have yielded a sample that was not fully representative of the intended audiences, which could have introduced bias. While our sample was both young and ethnically diverse, the absence of male participants means that a comprehensive set of perspectives was likely not obtained. Relatedly, the recruitment strategy relied on sampling a population of individuals with a history of engagement with *Youth Speaks* events. While this strategy aligns with the California Department of Public Health's expectations around the dissemination of this content, it likely means that the sample *a priori* had a positive inclination toward art as a means of communication, further limiting generalizability. However, the nature of some of the participants' comments regarding the relative (in)appropriateness of spoken word as a medium to communicate about health suggests that participants maintained a critical view of the potential role of art for this purpose. Fourth, because of time limitations, we could only present participants with specific content from each campaign, selected because it was felt to be representative of the larger content. The videos that were presented from each campaign, however, were designed for similar target audiences, respectively (Black and Latinx populations). Finally, our study did not assess the relative reach of the *Survival Pending Revolution* campaign. It is widely recognized that campaigns such as *The Conversation* require and receive significant investments and resources to ensure broad reach. Future comparative studies of reach would need to provide similar resources to any content that is the subject of such inquiry.

## Conclusion

Conveying public health information in ways that successfully engage target audiences and activate them to adhere to public health recommendations and guidance is a very challenging undertaking. This endeavor is especially challenging when the intended audiences come from marginalized communities with a history of structural oppression and a high prevalence of mistrust in public health authorities. This challenge is further amplified in the contemporary communication environment that often is awash with misinformation and disinformation (23). Overcoming these challenges will require bold and innovative communication strategies. Our analysis of the *Survival Pending Revolution* campaign suggests that there is a promising alternative strategy to advance public health communications among BIPOC communities—including, but not limited to COVID-19 vaccination. Such a strategy situates “non-experts,” as part of an inclusive “we,” to be the messengers who name the fact that health and resultant health inequities are a function of the ecologies in which we live (not just individual actions), who call for accountability of institutions in power, and who advocate for structural change—all in service of advancing the kind of collectivism and interconnectedness that is at the root of public health action. In this case, the stories that animate this campaign reflect the lived experiences of YOC poets who give voice to the realities and critical mindsets of those in their community.

The goal of critical communication approaches is to aid disparity populations in both resisting and navigating systems that continue to locate them on the margins of society. Our qualitative evaluation of content from the *Survival Pending Revolution* campaign suggests that an arts-based approach authored by the youth of color organically generated critical communications that explicitly situate COVID-19 vaccination decision-making within the broader context of the structural determinants of health that are at the root of health inequities. Our evaluation further suggests that a campaign aligned with critical communication theory, when compared to a well-funded, state-of-the-art public heath campaign, can promote message salience, enable emotional engagement and provide a form of validation among historically oppressed groups such that they may be more open to attending to, and potentially acting on, the health communications they are exposed to. Specifically, our evaluation provides preliminary evidence that—for marginalized populations—a critical approach to public health communication may generate superior degrees of engagement with, and openness to, evidence-based public health content when compared to more traditional approaches. Taken together, our research suggests that critical communication theory offers a promising formative and interventional approach to engendering trust in public health messaging and promoting health equity. Given the inconsistent and limited benefits of traditional public health communication in reaching marginalized populations, future work should rigorously examine the relative efficacy of content designed with critical communication approaches vs. more traditional approaches in terms of reach, engagement, and behavior change. In addition, given the complexity, ubiquity, and inconsistent reliability of public health communication content now available on the internet, additional studies should examine the effectiveness of critical communication approaches in combating misinformation and disinformation.

## Data availability statement

The raw data supporting the conclusions of this article will be made available by the authors, without undue reservation.

## Ethics statement

The studies involving human participants were reviewed and approved by UCSF IRB. Written informed consent for participation was not required for this study in accordance with the national legislation and the institutional requirements.

## Author contributions

All authors listed have made a substantial, direct, and intellectual contribution to the work and approved it for publication.

## References

[1] Official California State Government Website. California For All. Vaccination Data. Available online at: https://covid19.ca.gov/vaccination-progress-data/ (accessed February 2, 2023).

[2] Zoller HM Kline KN. Theoretical contributions of interpretive and critical research in health communication. In: Beck C editor. Communication Yearbook (Vol. 32). (New York: Routledge). (2008). p. 89–136. 10.1080/23808985.2008.11679076

[3] Kline KN Khan S. Doing critical health communication: negotiating the terrain of transdisciplinary collaboration. Front Commun Sec Health Commun. (2019) 4:51. 10.3389/fcomm.2019.00051

[4] Zoller HM. Critical health communication methods: challenges in researching transformative social change. Front Commun. (2019) 4:41. 10.3389/fcomm.2019.0004126189487

[5] Freedman DA Bess KD Tucker HA Boyd DL Tuchman AM Wallston KA. Public health literacy defined. Am J Prev Med. (2009) 446–51. 10.1016/j.amepre.2009.02.001 19362698

[6] Schillinger D Ling PM Fine S Boyer CB Rogers E Vargas RA. Reducing cancer and cancer disparities: lessons from a youth-generated diabetes prevention campaign. Am J Prev Med. (2017) 53:S103–S113. 10.1016/j.amepre.2017.05.024 28818240 PMC8491805

[7] Rogers EA Fine S Handley MA Davis H Kass J Schillinger D. Development and early implementation of the bigger picture, a youth-targeted public health literacy campaign to prevent type 2 diabetes. J Health Commun. (2014) 19:144–60. 10.1080/10810730.2014.940476 25315590 PMC4217646

[8] Abbs E Daniels R Schillinger D. Type 2 diabetes as a socioecological disease: can youth poets of color become messengers of truth and catalysts for change? Health Promot Pract. (2022) 23:583–93. 10.1177/15248399211007818 33989074 PMC8590708

[9] Freire P. Pedagogy of the Oppressed. New York: Herder and Herder. (1975).

[10] Boal A. The Aesthetics of the Oppressed. New York: Routledge Press. (2006). 10.4324/9780203969830

[11] Newton HP. To Die for the People: The Writings of Huey P. Newton New York: Random House. (1972) 104.

[12] Dada D Djiometio JN McFadden SM Demeke J Vlahov D Wilton L. Strategies that promote equity in COVID-19 vaccine uptake for black communities: a review. J Urban Health. (2022) 99:15–27. 10.1007/s11524-021-00594-3 35018612 PMC8751469

[13] Campbell KA Orr K Durepos E Nguyen L Li L Whitmore C. Reflexive thematic analysis for applied qualitative health research. Qual Report. (2021) 26:2011–28. 10.46743/2160-3715/2021.5010

[14] Schillinger D Tran J Fine S. Do low income youth of color see “the bigger picture” when discussing type 2 diabetes: a qualitative evaluation of a public health literacy campaign. Int J Environ Res Public Health. (2018) 15:840. 10.3390/ijerph15050840 29695114 PMC5981879

[15] Schillinger D., Huey N. Messengers of truth and health-young artists of color raise their voices to prevent diabetes. JAMA. (2018) 319:1076–8. 10.1001/jama.2018.0986 29450481

[16] Stuckey HL Nobel J. The connection between art, healing, and public health: A review of current literature. Am J Public Health. (2010) 100:254–63. 10.2105/AJPH.2008.156497 20019311 PMC2804629

[17] Chepp V. Activating politics with poetry and spoken word. Contexts. (2016) 15:42–7. 10.1177/1536504216685109

[18] Manresa GA Glăveanu V. Poetry in and for Society: Poetic Messages, Creativity, and Social Change. In: Lehmann O Chaudhary N Bastos AC Abbey E editor. Poetry and Imagined Worlds, (Palgrave Macmillan, Cham). (2017). p. 43–62. 10.1007/978-3-319-64858-3_3

[19] Alter C. The School Shooting Generation has had Enough Time. (2018). Available online at: https://time.com/longform/never-again-movement/ (accessed February 15, 2023).

[20] Alvarez N Mearns J. The benefits of writing and performing in the spoken word poetry community. The Arts in Psychotherapy. (2014) 41:263–8. 10.1016/j.aip.2014.03.004

[21] Tyson EH. Hip hop therapy: an exploratory study of a rap music intervention with at-risk and delinquent youth. J Poetry Therapy. (2002) 15:131–44. 10.1023/A:1019795911358

[22] Prendergast M. “Poem Is What?” poetic inquiry in qualitative social science research. Int Rev Qualitative Res. (2009) 1:541–68. 10.1525/irqr.2009.1.4.541

[23] Schillinger D Chittamuru D Ramírez AS. From “infodemics” to health promotion: a novel framework for the role of social media in public health. Am J Public Health. (2020) 110:1393–6. 10.2105/AJPH.2020.305746 32552021 PMC7427212

[24] Brinson NH. Resistance to persuasion: examining the influence of political ideology on COVID-19 vaccine uptake hesitancy. Front Commun. (2022) 6:760847. 10.3389/fcomm.2021.760847

[25] Adams SH Schaub JP Nagata JM Park MJ Brindis CD Irwin CE. Young adult perspectives on COVID-19 vaccinations. J Adolesc Health. (2021) 69:511–14. 10.1016/j.jadohealth.2021.06.003 34274212 PMC8277980

[26] Xu Y Margolin D Niederdeppe J. Testing strategies to increase source credibility through strategic message design in the context of vaccination and vaccine hesitancy. Health Commun. (2021) 36:11. 10.1080/10410236.2020.1751400 32308037

[27] Kuru O Stecula D Lu H Ophir Y Chan M-pS Winneg K. The effects of scientific messages and narratives about vaccination. PLoS ONE. (2021) 16:e0248328. 10.1371/journal.pone.0248328 33760856 PMC7990169

[28] Nyhan B Reifler J Richey S Freed GL. Effective messages in vaccine promotion: a randomized trial. Pediatrics. (2014) 133:e835–e842. 10.1542/peds.2013-2365 24590751

[29] Goldstein S MacDonald NE Guirguis S. SAGE working group on vaccine hesitancy. Health communication and vaccine hesitancy. Vaccine. (2015;) 33:4212–4. 10.1016/j.vaccine.2015.04.042 25896382

[30] Mohan J. Dutta-Bergman theory and practice in health communication campaigns: a critical interrogation. Health Commun. (2005) 18:103–122. 10.1207/s15327027hc1802_1 16083406

[31] Dawkins-Moultin L McDonald A McKyer L. Integrating the principles of socioecology and critical pedagogy for health promotion health literacy interventions. J Health Commun. (2016) 21: 30–35. 10.1080/10810730.2016.1196273 27668970

[32] Canfield KN Menezes S Matsuda SB Moore A Mosley Austin AN Dewsbury BM. Science communication demands a critical approach that centers inclusion, equity, and intersectionality. Front Commun. (2020) 5:2. 10.3389/fcomm.2020.00002

[33] Carter AL Alexander A. Soul food: [re]framing the african-american farming crisis using the culture-centered approach. Front Commun. (2020) 5:5. 10.3389/fcomm.2020.00005

[34] Hernández LH De Los Santos Upton S. Critical health communication methods at the US-Mexico border: violence against migrant women and the role of health activism. Front Commun. (2019) 4:34. 10.3389/fcomm.2019.00034

[35] Zoller HM. Health activism targeting corporations: a critical health communication perspective. Health Commun. (2017) 32:219–29. 10.1080/10410236.2015.1118735 27218457

[36] Sastry S Basu A. How to have (critical) method in a pandemic: outlining a culture-centered approach to health discourse analysis. Front Commun. (2020) 5:585954. 10.3389/fcomm.2020.585954

